# About Doctors

**Published:** 1869-07

**Authors:** 


### ABOUT DOCTORS.

ADVERTISING AND MEDICAL ETHICS.

The code of medical ethics, adopted by the American Medical Association, contains this clause:—"It is derogatory to the dignity of the profession to resort to public advertisements, or private cards, or handbills, inviting the attention of individuals affected with particular diseases; or to publish cases and operations in the daily prints, or suffer such publications to be made."

Physicians who belong to the class called "regulars," denounce every medical man as a charlatan, who violates this rule. Yet there is not a man engaged in the practice of medicine, who has attained any notoriety or has become at all eminent in his profession, who does not habitually violate this code.

From the "American Medical Association," down to the "Chemung County Medical Socity" we have a set of advertisers. The American Society have their poceedings published in the daily press of the city in which they convene, and the public are informed who read scientific papers upon different medical subjects, what physicians presented remarkable cases for examination before the grand body, who made speeches, and who the most eminent men of this grand body are, giving them all a handsome puff, and of course, so "inviting the attention of individuals affected with particular diseases to the physicians named.

County societies publish their entire proceedings in the daily papers, naming the physicians present, the titles of the papers presented, and by whom, the names of the officers elected, and an elaborate account of cases presented to the society by some physician fortunate enough to have a case to present.—Should a child fall from a swing and dislocate an arm, some how or other, the "local" hears of it, and invariably mentions the name of the attending physician. Is a limb amputated, the fact is made known and the public are not left to guess who the operator was. Should a lady be safely delivered of twins, the fact is noted and the name of the accoucher receives honorable mention. Indeed, we have numerous notices collected of the doings of every physician in this county, and some of those in neighboring counties, and yet, almost every one of them will tell you that an "advertising man" is a quack. Should one surgeon of a city have more surgical operations to perform than all the others combined, he of course will receive more "notices" through the *press*—hence more notoriety—more experience—more cash to pay the printer. It is expected that less enterprising physicians will have less to do, therefore more time to carp and grumble at the success of his superior. The old numb -skull who happens to secure a case and a notice occasionally, will of course cry "quack" to the physician who has them daily—it is to his interest to do so. If he can't secure patronage through his worthiness, he must freighten cases of the skillful physicians to him, by his favorite cry of quack! quack! If he meets your patients returning from your office, he intersepts them to warn them against you, not that he wants the case himself—O, no,—it is a duty he owes to his—pocket to say to them that you advertise, therefore are a quack, and will never cure them—never. But if they will examine the proceedings of the last medical society, published in the city papers, they will see that he is the author of an article on club-foot, or abortion, or something of that sort, and therefore is more competent to cure them than that miserable quack who is for ever receiving notices through the press.

The best guide for the public to judge of the worth of a physician, is not to listen to what his professional *brethren* say of him, but to observe his scholarly attainments, and to note his success in practice. If he receives more "notices" than those who call him "quack," it is more than likely that he receives more patronage, and is therefore better entitled to your confidence. If he is a specialist, devoting his time to a particular class of diseases, then is he all the more likely to be successful in the management of such diseases. Do not be frightened from him by what other physicians say of advertising. That dodge has long since been exploded, for every physician who *can*, will certainly advertise, and if he fails to report his cases, it is simply because he has none to report. *Everybody* advertises now-a-days, and no one is worthy of support that does not.

No intelligent physician will allow himself to be puffed—or will publish certificates of cure—that is abominable. But a modest card setting forth that he is devoting his attention to special diseases, is legitimate. Persons in search of such a physician will then know where to find him, and both will be mutually benefitted.

If the physician has an important surgical operation, and the press makes note of it without

*puffing* the Doctor, that is legitimate, eminently proper; it will refer others similarly afflicted to the operator, giving him larger experience, and so making him "a benefactor to the race."—*There is no physician, now living, who is eminent in his profession, that has not gained his notoriety by a judicious system of advertising!* Therefore is the clause from the code of medical ethics, above quoted a useless scare-crow.

